# Comparing Two Folate Receptor β‑Targeted Tracers in a Rat Model of Experimental Autoimmune Myocarditis

**DOI:** 10.1021/acsptsci.4c00749

**Published:** 2025-06-25

**Authors:** Erika Atencio Herre, Xiang-Guo Li, Heidi Liljenbäck, Senthil Palani, Putri Andriana, Arghavan Jahandideh, Jenni Virta, Imran Iqbal, Pyry Dillemuth, Jonne Kunnas, Maxwell W.G. Miner, Johan Rajander, Hasan Mansour A Mansour, Nathan A. Cleveland, Madduri Srinivasarao, Philip S. Low, Juhani Knuuti, Antti Saraste, Anne Roivainen

**Affiliations:** † Turku PET Centre, 8058 University of Turku, Turku FI-20520, Finland; ‡ Department of Chemistry, University of Turku, Turku FI-20500, Finland; § InFLAMES Research Flagship, University of Turku, Turku FI-20014, Finland; ∥ Turku PET Centre, Turku University Hospital, Turku FI-20520, Finland; ⊥ Turku Center for Disease Modeling, University of Turku, Turku FI-20520, Finland; # Accelerator Laboratory, Turku PET Centre, 1040 Åbo Akademi University, Turku FI-20520, Finland; ∇ Department of Chemistry, 311308 Purdue University, West Lafayette, Indiana 47907, United States; ○ Heart Centre, Turku University Hospital and University of Turku, Turku FI-20520, Finland

**Keywords:** experimental autoimmune myocarditis, folate, folate receptor β, myocarditis, PET

## Abstract

Folate receptor β (FR-β) expression may serve as a marker of activated macrophages involved in autoimmune myocarditis. The positron emission tomography (PET) tracer *N*-succinimidyl 4-[^18^F]­fluorobenzoate-conjugated folate ([^18^F]­SFB-FOL) effectively targets FR-β-positive macrophages in rheumatoid arthritis. Here, we examined [^18^F]­SFB-FOL for detecting myocardial inflammation via FR-β in a rat model of experimental autoimmune myocarditis (EAM), in comparison with the established FR-β-targeted PET tracer aluminum fluoride-18-labeled 1,4,7-triazacyclononane-1,4,7-triacetic acid-conjugated folate ([^18^F]­FOL). EAM was induced in 22 Lewis rats through cardiac myosin immunization. Rats underwent 2-deoxy-2-[^18^F]­fluoro-d-glucose ([^18^F]­FDG) PET to visualize myocardium, followed by dynamic PET with [^18^F]­SFB-FOL or [^18^F]­FOL at Days 14, 21, or 28 postimmunization. Postimaging, myocardial tissues were assessed by γ-counting, autoradiography, and CD68 immunohistochemistry to quantify macrophage presence. Both tracers showed high radiochemical purity and *in vivo* stability. Inflammation-rich myocardial lesions were confirmed, with macrophages occupying 9.9% ± 1.1 of the tissue area. PET imaging revealed significantly higher uptake of both tracers in inflamed myocardium versus remote areas, confirmed by histology and autoradiography. Lesion-to-remote uptake ratios were 5.7 ± 1.8 for [^18^F]­SFB-FOL and 3.8 ± 0.5 for [^18^F]­FOL. Blood clearance and renal excretion were rapid for both tracers. No significant differences were observed in tracer uptake or macrophage density between Days 21 and 28. [^18^F]­SFB-FOL is a suitable tracer for detecting active myocardial inflammation via FR-β in EAM and performs comparably to [^18^F]­FOL.

## Graphical abstract

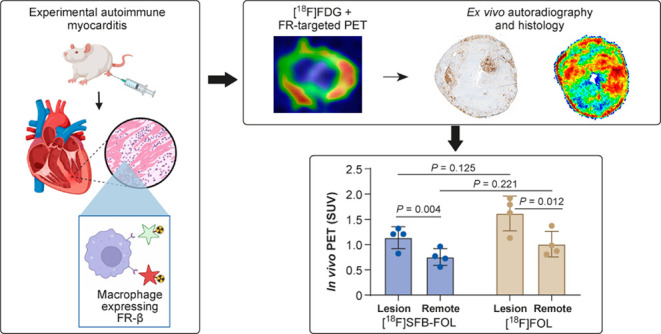

## Introduction

Myocarditis, a disease characterized by inflammation of the myocardium, is caused by several factors, including viral or bacterial infections, toxins, or inflammatory diseases such as sarcoidosis.^1^ Diagnosis can be challenging due to variability in clinical presentation. While endomyocardial biopsy is the most specific diagnostic method, its invasive nature, and variation in sensitivity depending on the area of myocardium sampled, have led to the use of noninvasive cardiac imaging for diagnosis.^2^ While 2-deoxy-2-[^18^F]­fluoro-d-glucose ([^18^F]­FDG) positron emission tomography (PET) is highly sensitive and specific for detecting active myocarditis, unsuccessful suppression of physiological glucose metabolism in healthy myocytes can reduce the specificity of the [^18^F]­FDG PET signal;^3–5^ therefore, more specific radiotracers are required for accurate diagnosis and monitoring of active inflammation in myocarditis.

Previously, we evaluated a folate receptor beta (FR-β)-targeting radiotracer, aluminum fluoride-18-labeled 1,4,7-triazacyclononane-1,4,7-triacetic acid conjugated folate ([^18^F]­FOL), in a rat model of experimental autoimmune myocarditis (EAM), and found specific uptake of the tracer in inflammatory lesions containing FR-β-positive macrophages.^6^ Furthermore, we demonstrated that FR-β-positive macrophages were present in human cardiac sarcoid lesions. In this study, we evaluated another FR-β-targeting radiotracer, N-succinimidyl 4-[^18^F]­fluorobenzoate-conjugated folate ([^18^F]­SFB-FOL), in the same EAM model. This tracer demonstrated high specific uptake in inflamed synovial tissues in another experimental model, as well as in patients with rheumatoid arthritis, with a low background signal and rapid clearance from the blood.^7,8^ Since [^18^F]­SFB-FOL has already been used in human trials, proven safe and well-tolerated,^8^ it paves the way for clinical trials in other indications, too. This study aims to evaluate the ability of [^18^F]­SFB-FOL to detect and monitor myocardial inflammation in the EAM model using PET/computed tomography (CT) imaging, and compare its performance to the previously established [^18^F]­FOL tracer. By performing this comparative study, we will gain valuable information on their PET imaging capabilities and biokinetic properties in the rat EAM model. Given that previous studies reported successful results in imaging inflammation, we hypothesized that [^18^F]­SFB-FOL might detect active inflammation in myocardial lesions as effectively as [^18^F]­FOL, making it a promising tracer for monitoring disease progression over time. These findings will be valuable for the design of our future clinical studies involving patients with autoimmune myocarditis.

## Materials and Methods

### Animal Model and Study Design

All animal experiments were approved by the National Project Authorisation Board in Finland (license number ESAVI/37123/2020), and were carried out in compliance with the relevant European Union Directive 2010/EU/63 on the protection of animals used for scientific purposes.

Thirty-one male Lewis rats (6–8 weeks old, weight 296 ± 33 g [range 217–439 g]) were used. To induce myocarditis, 22 rats were immunized with porcine cardiac myosin in complete Freund’s adjuvant as described previously.^6^ Immunizations were performed under isoflurane anesthesia, and buprenorphine was given afterward for pain management. All animals had access to food and water *ad libitum*.

The first aim of the study was a head-to-head comparison of [^18^F]­SFB-FOL and [^18^F]­FOL, evaluating their uptake in inflamed myocardium using *in vivo* PET/CT at 20 and 21 days postimmunization (*n* = 4). To further investigate myocardial uptake and biodistribution of both tracers, digital autoradiography and *ex vivo* γ-counting were performed in additional animals at 21 days postimmunization (*n* = 7 and *n* = 4, respectively). The specificity of [^18^F]­SFB-FOL uptake in inflamed myocardium of immunized rats preinjected with a 100-fold molar excess of folate glucosamine before [^18^F]­SFB-FOL administration (*n* = 7) was evaluated using PET/CT (*in vivo* data), and in tissue binding and cell binding studies (*in vitro* data). Finally, [^18^F]­SFB-FOL was used to evaluate disease progression on Days 14, 21, and 28 postimmunization (*n* = 4). Nine Lewis rats (*n* = 5 [^18^F]­SFB-FOL, *n* = 4 [^18^F]­FOL) served as healthy controls to evaluate *in vivo* stability and biodistribution. The study design is shown in Figure 1.

**Figure 1:**
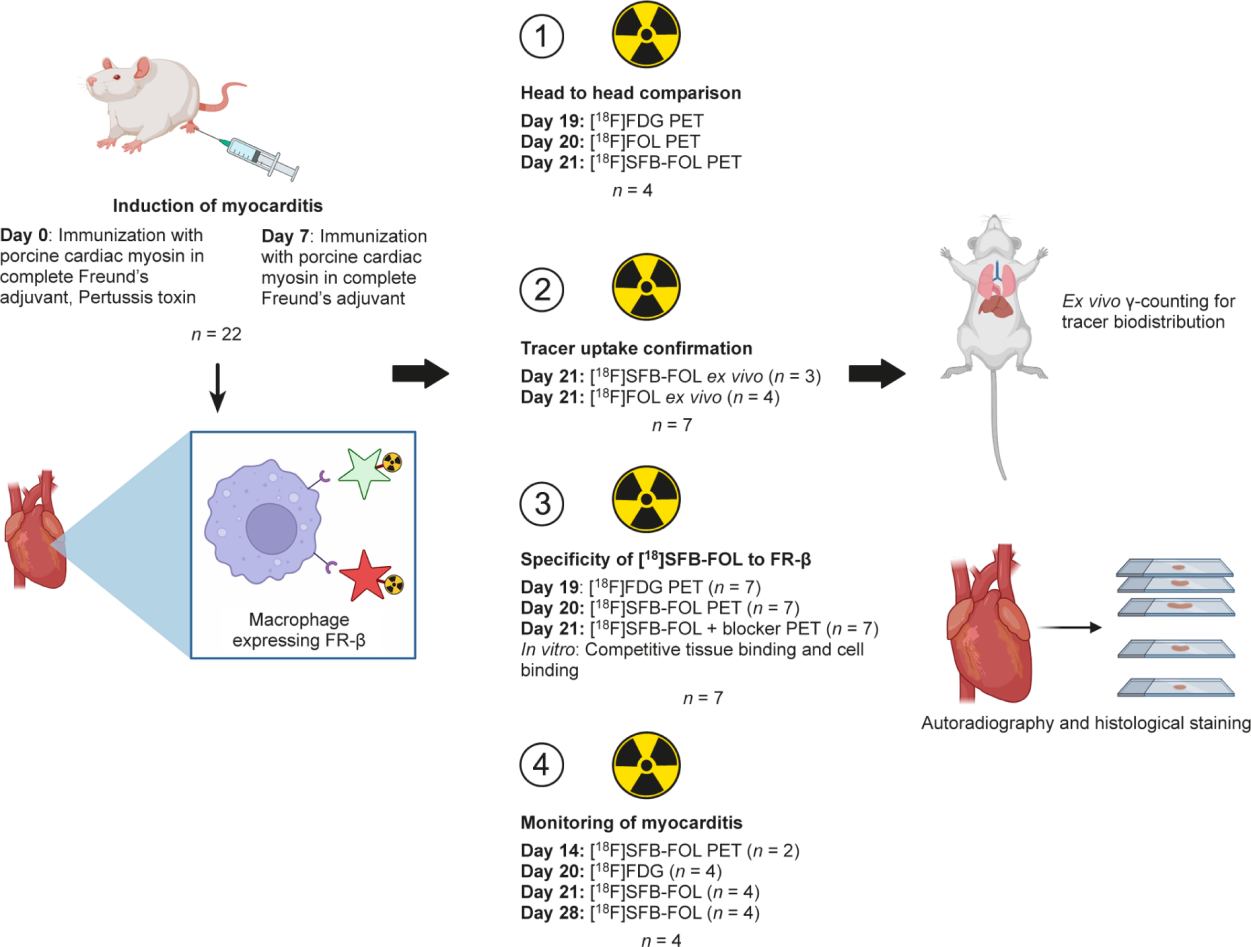
Study design (Created in BioRender. Atencio, E. (2025) https://BioRender.com).

### Radiochemistry

Chemical structure of the ^18^F-labeled folate-based tracers used in the study are shown in Figure S1. [^18^F]­SFB-FOL was produced as previously described.^7^ Briefly, [^18^F]­SFB was first synthesized and purified using high-performance liquid chromatography with radioactivity detection (radio-HPLC), and then conjugated to a folate precursor to prepare [^18^F]­SFB-FOL. The folate-NOTA conjugate and [^18^F]­FOL were produced as described previously.^9,10^ Good Manufacturing Practice-grade [^18^F]­FDG was produced at the Turku PET Centre. The radiochemical purity of the tracers was determined by radio-HPLC.

The *in vitro* stability of [^18^F]­SFB-FOL in the end product formulation was monitored by keeping the product at room temperature (RT) for 4 h, sampling hourly for radio-HPLC analysis. The lipophilicity of [^18^F]­SFB-FOL was determined by calculating the distribution coefficient (Log*D*) in a mixture of 1-octanol and phosphate-buffered saline (PBS, pH 7.4).

### *In Vivo* PET/CT and Image Analysis

*In vivo* imaging was performed under isoflurane anesthesia using an Inveon Multimodality PET/CT (Siemens Medical Solutions). All animals (*n* = 15) underwent 60 min dynamic PET, starting from folate tracer injection. CT was performed for attenuation correction and myocardium anatomy was referenced via 10 min static PET acquired 20 min after [^18^F]­FDG injection a day earlier. The injected radioactivity doses were 43.7 ± 10.8 MBq (552.5 ± 263 μL) for [^18^F]­SFB-FOL, 50.3 ± 1.4 MBq (210 ± 140 μL) for [^18^F]­FOL, and 33.9 ± 2.7 MBq (232 ± 84 μL) for [^18^F]­FDG. For *in vivo* blocking studies, preinjection of a 100-fold molar excess of folate glucosamine (C_25_H_30_N_8_O_10_, 150–440 μL) was performed 15 min prior to tracer injection. PET data for the folate tracers were reconstructed using three-dimensional ordered-subset expectation maximization (2 iterations) with maximum a posteriori, using a shifted Poisson distribution (18 iterations, 16 subsets) algorithm (6 × 10 s, 4 × 60 s, 5 × 300 s, and 3 × 600 s time frames).

Carimas 2.10 software (Turku PET Centre) was used to construct polar maps of [^18^F]­SFB-FOL or [^18^F]­FOL uptake in the myocardium of the left ventricle (LV); the maps were generated based on myocardial contours and sampling points matching to coregistered [^18^F]­FDG images. The procedures are described in detail in the Supporting Information.^11^ Tracer uptake in segments with inflammatory lesions and in segments remote from inflamed lesions (as defined by later hematoxylin-eosin (H&E) staining) was measured from 20 to 40 min postinjection. Additional manually defined ROIs included the LV blood pool, the liver, the lungs, the front leg muscle, and the kidneys, all of which were evaluated from 20 to 40 min postinjection. To create time-activity curves, manual ROIs were also defined in myocardial inflammatory lesions and remote myocardium. The results are expressed as the mean standardized uptake value (SUV), normalized to body weight and total radioactivity injected, and as the percentage of injected radioactivity dose per cubic centimeter (%ID/cm^3^).

### *Ex Vivo* Biodistribution, Autoradiography, and Blood Analyses

Animals were sacrificed after final imaging at 70 min postinjection, and tissues of interest were excised, weighed, and radioactivity measured in a γ-counter (Triathler, Hidex). The results are expressed as the percentage of injected radioactivity dose per gram of tissue (%ID/g). Hearts were frozen and sectioned from base to apex (serial transverse 20-μm and 8-μm cryosections for autoradiography and histological staining). Slides were placed against an imaging plate (BAS-TR2025, Fujifilm) and exposed for at least 4 h, after which the imaging plates were scanned with a BAS-5000 Analyzer (Fujifilm) with 25 μm resolution.

Myocardial cryosections were stained with H&E for histological confirmation of lesions, and adjacent cryosections were stained immunohistochemically with an anti-rat CD68 antibody as described previously^6^ to confirm the presence of macrophages. The stained sections were scanned with a Pannoramic P1000 slide scanner (3DHistech). Tracer binding was assessed using TINA 2.1 (Raytest Isotopemessgeräte), GIMP 2.0 (www.gimp.com), and Pannoramic Viewer (3DHistech) softwares. Co-registration of digital autoradiography sections with corresponding H&E and CD68 staining was done using Carimas 2.10 software (Turku PET Centre) to visually confirm the overlap of tracer uptake from autoradiography to inflammatory myocardial lesions and macrophage presence.

To assess stability, blood cell binding, and plasma protein binding by [^18^F]­SFB-FOL and [^18^F]­FOL, blood samples were drawn from healthy controls 10–60 min postinjection. In immunized rats, plasma protein binding and stability were assessed in samples taken 70 min postinjection. Plasma and blood cells were separated by centrifugation, and plasma proteins were precipitated with acetonitrile before separation by centrifugation. Radioactivity in the whole blood, separated blood cells, plasma, separated plasma proteins, and the remaining plasma supernatant was measured in a γ-counter (1480 Wizard; PerkinElmer/Wallac). The plasma supernatant was analyzed by radio-HPLC using a semipreparative C18 column (Jupiter Proteo; 4 μm, 90 Å, 250 × 10 mm; Phenomenex).

### *In Vitro* Experiments

To determine the specificity of [^18^F]­SFB-FOL binding to FR-β, 20-μm myocardium cryosections from six immunized rats were used in an *in vitro* competitive binding study. Adjacent sections were separated into total binding and competitive binding groups. All slides were preincubated in PBS and then incubated in PBS with 247 ± 56 kBq (*n* = 4) of [^18^F]­SFB-FOL, either alone or with 100-fold molar excess of folate glucosamine (1 nM). After washing, radioactivity was detected by digital autoradiography, followed by tissue staining as described above.

To further confirm the binding specificity and affinity of [^18^F]­SFB-FOL for FR-β, *in vitro* studies were performed using Chinese hamster ovary (CHO) cells stably expressing human FR-β (CHO-FR-β^+^) or not (i.e., FR-β-negative cells; CHO-FR-β^–^).^12^ Briefly, cells were analyzed by flow cytometry to verify expression of FR-β.^13^ The CHO-FR-β^+^ and CHO-FR-β^–^ cells were cultured and allowed to attach to opposite sides of a Petri dish according to the LigandTracer (Ridgeview Instruments) guidelines. Cells were starved of folate and then incubated with increasing amounts of [^18^F]­SFB-FOL (15–45 nM). Tracer binding was measured using a LigandTracer Yellow instrument (Ridgeview Instruments); the assay protocol included sequential radioactivity measurements from the target cells (CHO-FR-β^+^), negative control cells (CHO-FR-β^–^), and the cell-free background regions on the Petri dish. The ratio of bound [^18^F]­SFB-FOL (to the cells) to background (Petri dish), and the K_*D*_ value, were determined using TraceDrawer software (Ridgeview Instruments).

### Statistical Analysis

Data are presented as the mean ± standard deviation (SD). Student’s *t*-tests, both paired and unpaired, were used to compare data, and a mixed-effects model was used to compare ROI uptake over time in the longitudinal study (due to missing values at one time point). The threshold for statistical significance was *p* < 0.05.

## Results

### Radiochemistry and Tracer Characteristics

Both tracers were produced with high radiochemical purity ([^18^F]­SFB-FOL = 97.4% ± 2.5, *n* = 22; [^18^F]­FOL = 88.3% ± 9.3, *n* = 4; *p* = 0.008). The average decay-corrected radiochemical yield of [^18^F]­SFB-FOL was 11.4% ± 7.7 and 19.5% ± 6.9 for [^18^F]­FOL (*p* = 0.068). [^18^F]­SFB-FOL had high molar activity (45.2 ± 22.8 GBq/μmol, *n* = 3). In the formulation medium, [^18^F]­SFB-FOL remained stable at RT, with a purity of 94% after 4 h (compared with initial 100% purity). The distribution coefficient (Log*D*, 1-octanol/PBS, pH 7.4) of [^18^F]­SFB-FOL was −2.0 ± 0.4 (*n* = 3), indicating high hydrophilicity. The tracer characteristics are shown in Table 1.

**Table 1:** Tracer Characteristics

	[^18^F]SFB-FOL	[^18^F]FOL
Molecular formula	C_36_H_54_FN_10_O_12_	C_37_H_51_AlFN_12_O_12_
Molecular weight	818.87 g/mol	899.86 g/mol
Radiochemical purity	97.4% ± 2.5 (*n* = 22)	88.3% ± 9.3 (*n* = 4)
Molar activity	45.2 ± 22.8 GBq/μmol (*n* = 3)	77 ± 22 GBq/μmol^a^
Lipophilicity (Log*D*)	–2.0 ± 0.4 (*n* = 3)	–3.3 ± 0.4^a^
Plasma protein binding	8.5% ± 1.0 (*n* = 4)†	24.5% ± 16.3 (*n* = 3)†
	13.9% ± 1.9 (*n* = 5)‡	26.5% ± 2.7 (*n* = 4)‡
Blood cell binding	22.0% ± 2.1 (*n* = 4)†	14.3% ± 9.0 (*n* = 3)†
*In vivo* stability	84.6% ± 8.8 (*n* = 4)†	91.0% ± 2.4 (*n* = 3)†
	57.4% ± 24.2 (*n* = 4)‡	88.6% ± 6.1 (*n* = 4)‡

a Reprinted with permission of Silvola et al. Measurements were taken at 60 min postinjection of †healthy Lewis rats and ‡immunized rats.

In healthy control animals, the average plasma protein binding by [^18^F]­SFB-FOL 60 min postinjection was 9.1% ± 1.0 (*n* = 4), compared with 24.5% ± 16.3 for [^18^F]­FOL (*n* = 3; *p* = 0.173). There was no significant difference with respect to blood cell binding ([^18^F]­SFB-FOL (22.0% ± 2.1) and [^18^F]­FOL (14.3% ± 9.0%, *p* = 0.219)). Evaluation of tracer plasma protein binding in rats yielded the following: 13.9% ± 1.9 (*n* = 5) for [^18^F]­SFB-FOL and 26.5% ± 2.7 for [^18^F]­FOL (*n* = 4; *p* = 0.0002). Both tracers demonstrated good *in vivo* stability, with 84.6% ± 8.8 of [^18^F]­SFB-FOL and 91.0% ± 2.4 of [^18^F]­FOL remaining intact in blood plasma 60 min postinjection (*p* = 0.347). HPLC detected radiometabolites derived from both tracers (representative chromatograms of [^18^F]­SFB-FOL in Figure S2). The *ex vivo* biodistribution of tracers in healthy Lewis rats at 70 min post-Injection is shown in Table S1. When evaluating [^18^F]­SFB-FOL *ex vivo* biodistribution in healthy and immunized EAM rats, we saw tracer uptake was significantly higher in a few organs of EAM rats, including the heart, spleen, thymus, and liver, compared to healthy rats (*p* = 0.006, 0.003, 0.002, and 0.006, respectively, Table S2).

### Analysis of Autoimmune Myocarditis in Rats

All immunized rats (*n* = 22) had myocardial inflammatory lesions located in the LV or in the free wall of the right ventricle. Eighteen rats had lesions throughout the thickness of the myocardium, whereas three showed mainly pericardial inflammation extending only to the adjacent myocardium. Localization of inflammatory lesions was verified by H&E staining and immunohistochemical staining with an anti-CD68 antibody. CD68-positive cells comprised 11.9% ± 2.6 (*n* = 17) and 11.8% ± 1.5 (*n* = 4, *p* = 0.944) of the total myocardial area at 21 and 28 days postimmunization, respectively.

### Comparison of [^18^F]­SFB-FOL and [^18^F]­FOL for Detection of Myocarditis

*In vivo* imaging of both folate tracers in the same animals, i.e., a head-to-head comparison, showed significantly higher uptake of both tracers in myocardial inflammatory lesions than in remote myocardium ([^18^F]­SFB-FOL lesion-to-remote ratio = 1.5 ± 0.1 *p* = 0.004; [^18^F]­FOL = 1.6 ± 0.2, *p* = 0.012, Figure 2 and Table 2).

**Figure 2:**
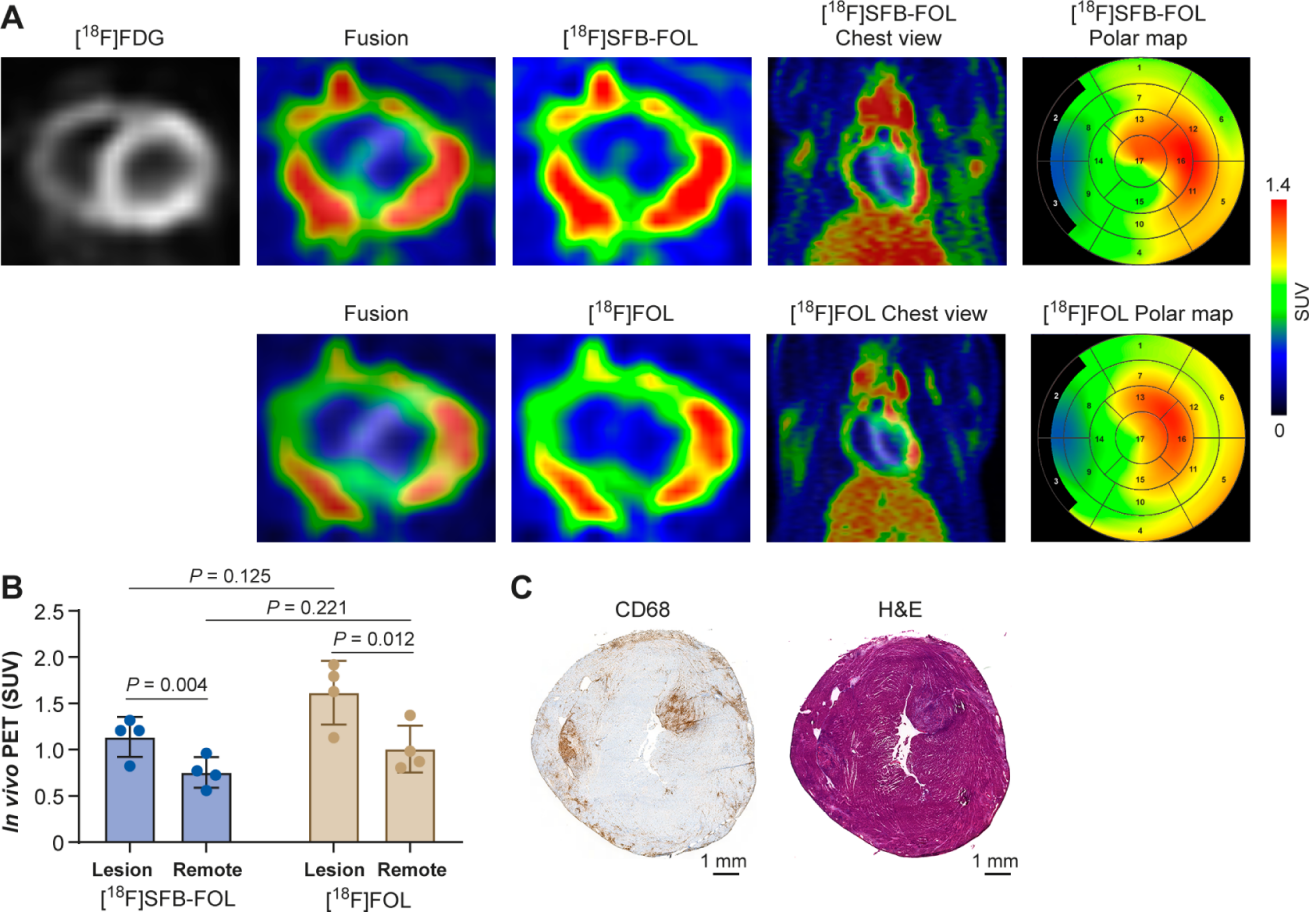
(A) Representative PET images and polar maps of a rat heart showing uptake and colocalization of both folate tracers with widespread inflamed myocardial lesions in the anterolateral wall of the left ventricle and the right ventricular free wall. (B) Comparison of tracer uptake in inflamed lesions (Lesion) versus myocardium remote from the lesions (Remote). (C) Anti-CD68 (macrophages) and hematoxylin-eosin (H&E) staining.

**Table 2:** *In Vivo* Uptake of Folate-Tracers 21 Days after Immunization by Myocardium and Adjacent Tissues at 20–40 Min Postinjection^a^

Region of interest	[^18^F]SFB-FOL	[^18^F]FOL	*P* value
Lesion (SUV_mean_)	1.1 ± 0.2	1.6 ± 0.3	0.125
Lesion-to-remote	1.5 ± 0.1	1.6 ± 0.2	0.521
Lesion-to-blood	3.4 ± 0.7	4.4 ± 1.0	0.148
Lesion-to-liver	0.7 ± 0.3	1.0 ± 0.2	0.198
Lesion-to-lung	4.8 ± 1.8	5.5 ± 2.2	0.438

a Results are expressed as the lesion mean standardized uptake value (SUVmean) and tracer uptake ratios between the lesion and other regions of interest; (mean ± SD, *n* = 4). *p* values calculated using Student’s *t* test.

Both tracers showed similar *in vivo* PET uptake patterns in inflamed lesions and in adjacent tissues (Table 2 and Figure S2). For [^18^F]­SFB-FOL, the ratio of uptake by inflamed myocardial lesions versus other selected tissues were as follows: 3.4 ± 0.7 vs the blood pool (*p* = 0.006), 0.7 ± 0.3 vs the liver (*p* = 0.152), and 4.8 ± 1.8 vs the lungs (*p* = 0.008). Similarly, the ratios for [^18^F]­FOL were 4.4 ± 1.0 vs the blood pool (*p* = 0.001), 1.0 ± 0.2 vs the liver (*p* = 0.732), and 5.5 ± 2.2 vs the lungs (*p* = 0.005). There were no significant differences in these uptake ratios between the tracers.

Digital autoradiography confirmed these results: both tracers exhibited significantly higher uptake in inflammatory lesions than in myocardium remote from the lesions (Figure 3). Binding of [^18^F]­SFB-FOL was 5.7 times higher in lesions than in remote myocardium, whereas that of [^18^F]­FOL was 3.7 times higher in lesions than in remote myocardium.

**Figure 3:**
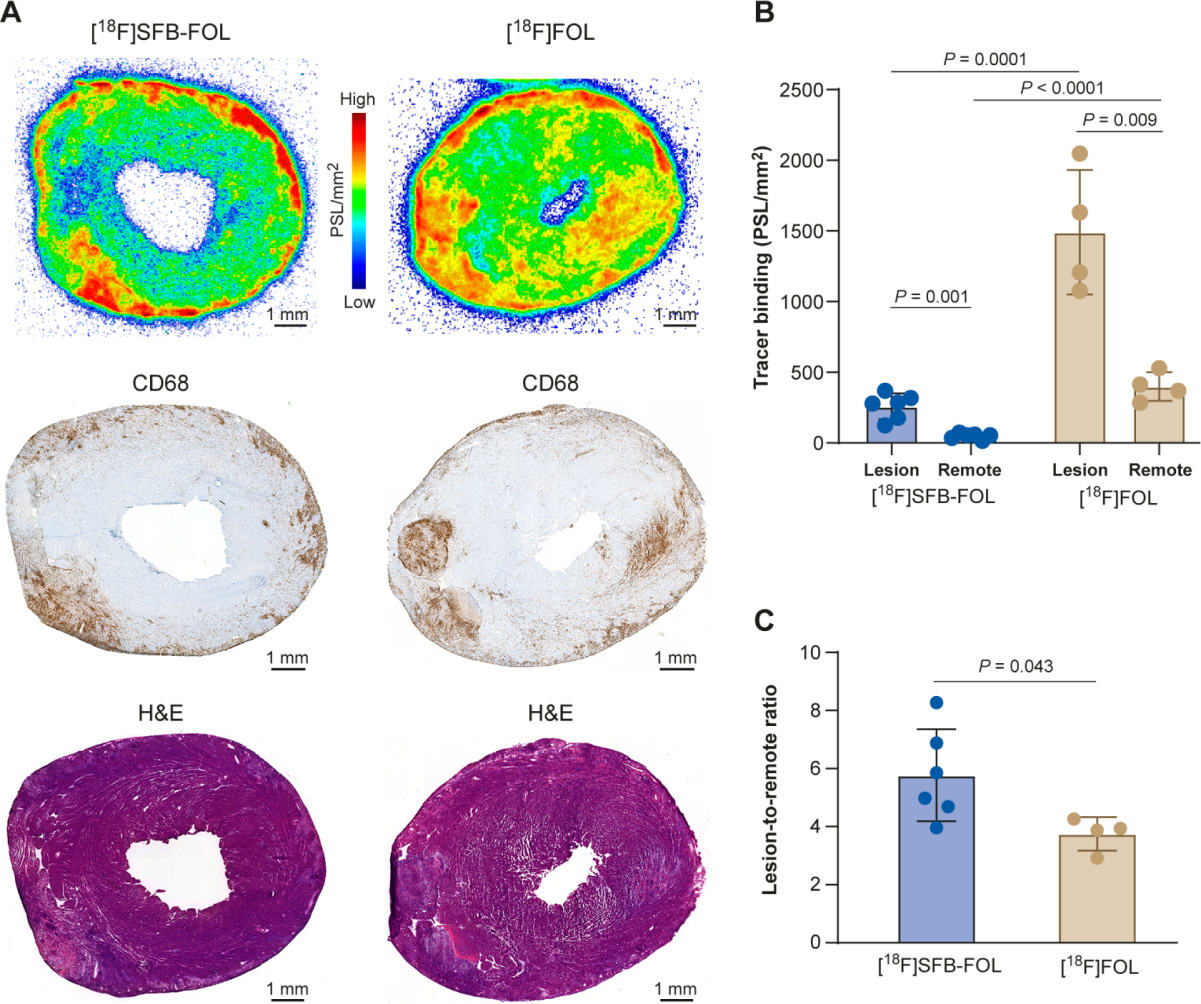
(A) *Ex vivo* autoradiographs of rat heart sections showing uptake of tracers in inflamed lesions containing CD68-positive macrophages. (B) Absolute binding of [^18^F]­FOL is higher than that of [^18^F]­FSB-FOL, but (C) the ratio of binding to inflammatory lesions and myocardium outside of the lesions (*i.e*., the lesion-to-remote ratio) is similar. PSL = photostimulated luminescence.

Both tracers cleared quickly from the blood, and showed higher uptake in lesions compared with myocardium outside the lesions or the blood pool. The average SUV in lesions from 20 to 40 min was 1.6 ± 0.2 for [^18^F]­SFB-FOL and 2.1 ± 0.0 for [^18^F]­FOL (*p* = 0.242), whereas those in remote myocardium were 0.5 ± 0.0 for [^18^F]­SFB-FOL and 0.7 ± 0.0 for [^18^F]­FOL (*p* = 0.139, Figure S4). When evaluating the *ex vivo* biodistribution of both tracers in animals, we noted several differences; most notably, uptake of [^18^F]­SFB-FOL in the liver and kidneys was significantly lower (*p* < 0.001 for both, Table S3). High *in vivo* and *ex vivo* uptake in kidneys indicated rapid excretion of the tracers via kidneys in the urine.

### FR-β Binding Specificity

Evaluation of the *in vivo* specificity of [^18^F]­SFB-FOL revealed a significant reduction in tracer uptake by inflammatory myocardial lesions after administration of a folate glucosamine (SUV = 0.5 ± 0.1 before vs 1.0 ± 0.4 after, *p* = 0.030, Figure 4b). The results also suggested significant blocking effects, as evidenced by reduced tracer uptake in all organs except blood and urine (Table S4).

**Figure 4:**
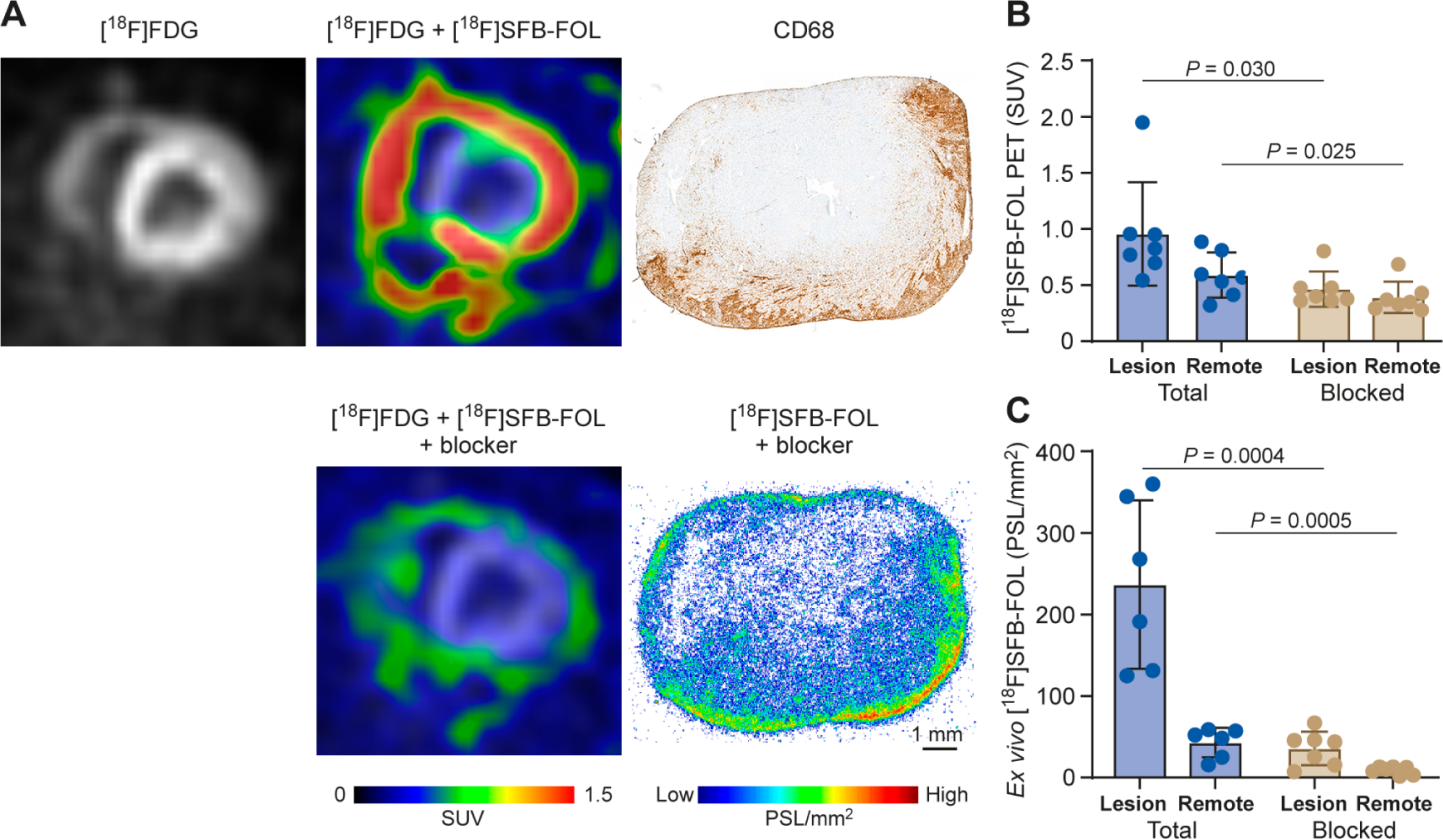
(A) Representative PET images of a rat heart showing uptake of [^18^F]­SFB-FOL and [^18^F]­SFB-FOL after coinjection of folate glucosamine (blocker), and a representative autoradiograph after imaging of [^18^F]­SFB-FOL and the blocker. The tracer uptake was predominantly found in regions containing CD68-positive macrophages within inflamed myocardial lesions. Quantification of [^18^F]­SFB-FOL uptake by (B) *in vivo* PET and (C) *ex vivo* autoradiography under total ([^18^F]­SFB-FOL) (Total) and blocked ([^18^F]­SFB-FOL with folate glucosamine) conditions in inflamed lesions (Lesion) versus myocardium outside of the lesions (Remote).

The tracer specificity was further supported by *in vitro* tissue studies, which revealed significantly lower binding of [^18^F]­SFB-FOL in myocardial lesions under competitive binding conditions versus normal conditions (17.6 ± 15.9 vs 269.7 ± 74.2 PSL/mm^2^, respectively; *p* < 0.0001, Figure S5). Additionally, the *in vitro* cell studies showed that [^18^F]­SFB-FOL had a binding affinity of 2.3 ± 0.6 nM for CHO-FR-β^+^ cells, with no clear binding to CHO-FR-β^–^ cells (Figure 5).

**Figure 5:**
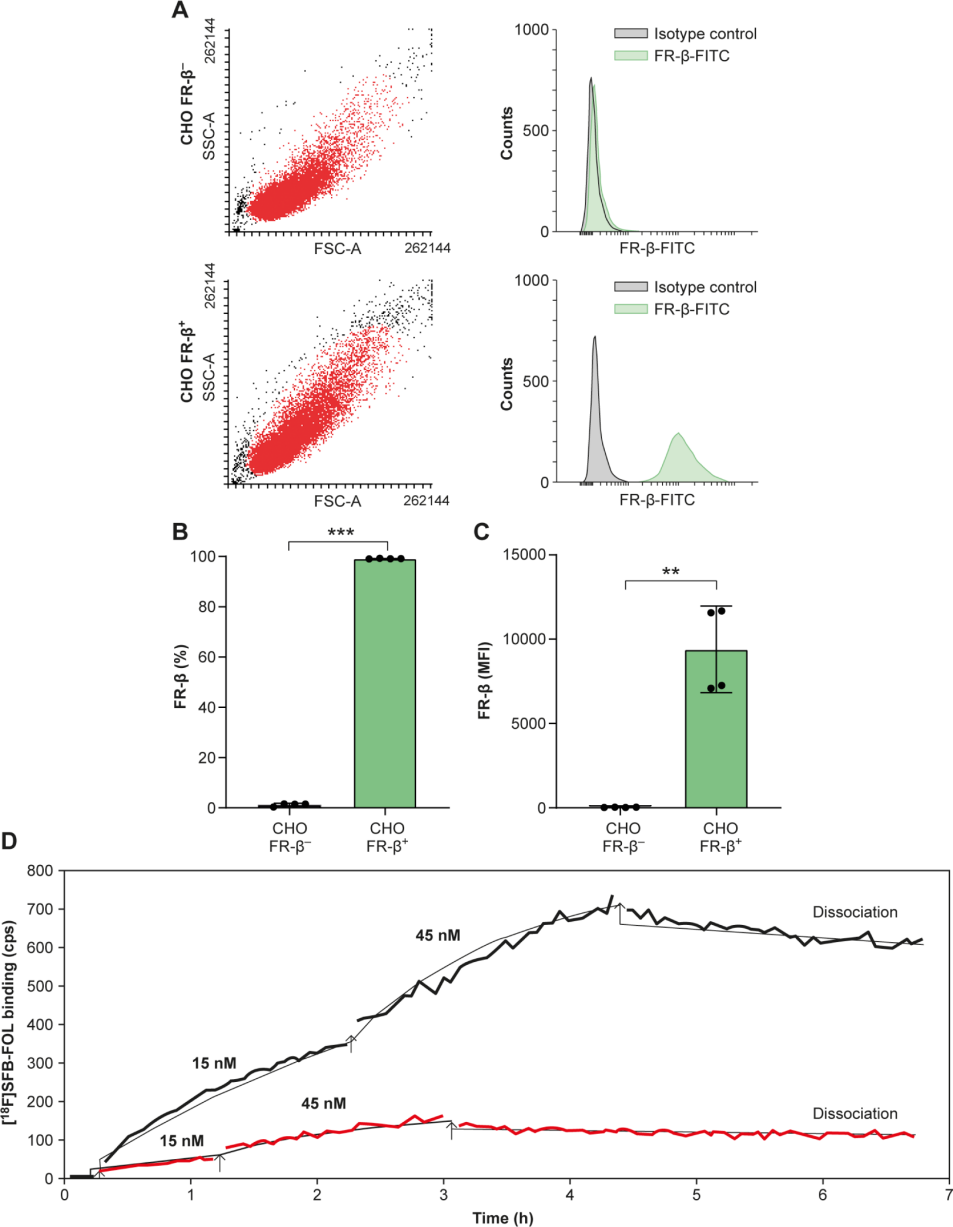
[^18^F]­SFB-FOL cell binding studies. (A) Representative flow cytometry plots of FR-β expression on CHO-FR-β and CHO-FR-β^–^ cells. SSC-A = side scatter area; FSC-A = forward scatter area; FITC = fluorescein isothiocyanate. Quantification of FR-β expression as (B) a percentage and (C) mean fluorescence intensity (MFI). (D) Real-time binding of [^18^F]­SFB-FOL, shows raw radioactivity counts per second (cps) after correction for the background signal and radioactive decay.

### Longitudinal Myocarditis Study Using [^18^F]­SFB-FOL

We found no significant differences in myocardial uptake of [^18^F]­SFB-FOL in inflammatory lesions at 14, 21, and 28 days postimmunization (lesion SUV 2.3 ± 1.5, 2.1 ± 1.1, and 1.5 ± 0.42, respectively; average %ID/cm^3^ = 0.9 ± 0.6, 0.8 ± 0.4, and 0.5 ± 0.1 respectively, *p* > 0.050 (comparing all groups); Figure S6). Additionally, there was no significant difference in any manually defined *in vivo* ROIs, *ex vivo* biodistribution, or number of CD68-positive cells in the myocardium between Days 21 and 28 (Tables S5 and S6, Figure S6). Analysis of digital autoradiographs obtained between Days 21 and 28 showed no significant differences in tracer uptake within inflammatory lesions, or in remote tissue (inflammatory lesions 258.5 ± 29.9 PSL/mm^2^ vs 298.8 ± 76.5 PSL/mm^2^, respectively, *p* = 0.508; remote tissues = 48.3 ± 13.7 PSL/mm^2^ vs 40.0 ± 20.2 PSL/mm^2^, respectively, *p* = 0.563).

## Discussion

Here, we demonstrate that uptake of [^18^F]­SFB-FOL in an EAM model is similar to that of the previously described [^18^F]­FOL,^6^ with high target-to-background ratios in macrophage-rich myocardial lesions. We also observed highly specific binding of [^18^F]­SFB-FOL to FR-β both *in vivo* and *in vitro*. Finally, we used [^18^F]­SFB-FOL to evaluate disease progression, finding no significant differences in the degree of inflammation (i.e., [^18^F]­SFB-FOL uptake) between time points.

Previously, [^18^F]­SFB-FOL was used to visualize arthritis, showing specific uptake in inflamed areas with high FR-β expression.^7,8^ It has also been studied for its ability to assess therapeutic response in an arthritis rat model with successful results.^14–16^ In this study, we aimed to visualize the inflamed myocardium via FR-β-expressing macrophages in an EAM model, and then compare [^18^F]­SFB-FOL with another FR-β-targeting radiotracer studied previously in the same EAM model.^6^ We found that, like [^18^F]­FOL, [^18^F]­SFB-FOL showed increased uptake in inflammatory myocardial lesions, and was predominantly found in regions containing macrophages that express FR-β.^6^ As in the previous study evaluating [^18^F]­FOL in this EAM model, [^18^F]­SFB-FOL showed a high target-to-background on both *in vivo* PET and *ex vivo* digital autoradiography. There were no differences in tracer uptake in tissues adjacent to the myocardium at the time of *in vivo* imaging, with both tracers showing SUVs in the LV blood, lungs, and liver that were comparable with those obtained in the previous study. Differences in renal radioactivity concentration and hepatic radioactivity uptake kinetics did not affect the assessment of myocarditis and can be explained by differences in plasma protein binding. Furthermore, we showed that binding of [^18^F]­SFB-FOL to FR-β *in vitro* was highly specific.

Previous studies using [^18^F]­FDG and [^11^C]­methionine in the same EAM model reported inflammatory-to-noninflammatory area ratios of 3.4 ± 0.7 and 2.1 ± 0.2, respectively.^17^ Our ratios were 1.5 ± 0.1 for [^18^F]­SFB-FOL and 1.6 ± 0.2 for [^18^F]­FOL. This difference can be attributed to our image analysis method. We used myocardial contours from coregistered [^18^F]­FDG images to create polar maps, rather than manually defined ROIs based solely on observed increases in the PET signal.^6,17^ While our current analysis method was necessary for unbiased comparison of [^18^F]­SFB-FOL and [^18^F]­FOL, it results in dilution of the signal by including noninflamed myocardium within each segment, leading ultimately to lower uptake ratios. However, the manually defined ROIs for the time-activity curves were comparable with those in our previous [^18^F]­FOL study (2.1 ± 1.1 in lesions, 0.4 ± 0.1 in remote tissue).^6^ Previous studies show that mRNA cytokine expression in the experimental myocarditis model distinguishes initial pro-inflammatory phase (14 days) from the recovery phase (25 days) postimmunization.^18^ The model has also been used to study uptake of [^18^F]­FDG for 4 weeks postimmunization, noting an increase in tracer uptake at 3 weeks (21 days) postimmunization (0.82 ± 0.3 %ID/cm^3^) and a decrease at 4 weeks (28 days) postimmunization (0.32 ± 0.1 %ID/cm^3^).^19^ It was therefore of interest to evaluate the utility of [^18^F]­SFB-FOL for monitoring disease progression because [^18^F]­FDG can be taken up by normal myocytes. When using [^18^F]­SFB-FOL to monitor uptake by myocardial lesions at various time points, we observed a pattern similar to that of [^18^F]­FDG; there were no significant differences in uptake of either tracer or the macrophage area.

The study has some limitations. Given the complexity of [^18^F]­SFB-FOL synthesis, we experienced frequent technical challenges including synthesis failures and low yields, which contributed to lower animal numbers on Day 14 of the longitudinal study. Given the longitudinal study design with the same animals imaged at all three points, we did not collect tissues or perform detailed histology at all three time points to more closely assess temporal changes of the disease or the expression of FR-β over time. The polar maps analysis method used for analyzing myocardial uptake in PET only provides information about uptake in the LV; therefore, it did not account for radiotracer uptake in the right ventricle despite clear inflammatory lesions being present in the histology of several animals. Furthermore, only male rats were used for the study; therefore, it remains to be seen whether the radiotracers would behave similarly, or whether the disease progression would vary, in females. Finally, the radio-metabolites derived from both tracers have not been identified; these are still to be studied.

## Conclusion

Inflamed myocardium in the rat EAM model can be detected specifically via FR-β-expressing macrophages using [^18^F]­SFB-FOL PET, which shows *in vivo* imaging characteristics similar to those of [^18^F]­FOL.

## Supplementary Material



## Data Availability

The original data of the work can be obtained from Prof. Anne Roivainen upon rational request.

## Notes

Detailed protocols for animal immunizations, tracer radiosynthesis, Log*D* determination, blood stability assays, image analysis procedures, and *in vitro* experiments. Supplementary figures present chemical structures of the radiotracers, HPLC chromatograms of radiometabolites from *in vivo* stability analysis, whole-body *in vivo* PET/CT images of both radiotracers, time-activity curves, results from competitive binding assays, and data from the longitudinal study. Supplementary tables provide biodistribution data comparing tracers in control versus immunized animals, results under blocking conditions, and additional findings from the longitudinal study (PDF)
