# Supplementary material for: Comparing Two Folate Receptor β‑Targeted Tracers in a Rat Model of Experimental Autoimmune Myocarditis
Source: ACS Pharmacol Transl Sci. 2025 Jun 25;8(10):3453–62. doi: 10.1021/acsptsci.4c00749 (PMC12519279; doi:10.1021/acsptsci.4c00749)
Supplement: Supplementary file 1 [file pt4c00749_si_001.pdf]

Supporting information

Comparing two folate receptor **β**-targeted tracers in a rat model of experimental autoimmune myocarditis

Erika Atencio Herre[1], Xiang-Guo Li[1,2,3,4], Heidi Liljenbäck[1,5], Senthil Palani[1], Putri Andriana[1], Arghavan Jahandideh[1], Jenni Virta[1], Imran Iqbal[1], Pyry Dillemuth[1], Jonne Kunnas[1], Maxwell W.G. Miner[1], Johan Rajander[6], Hasan Mansour A Mansour[7], Nathan A. Cleveland[7], Madduri Srinivasarao[7], Philip S. Low[7], Juhani Knuuti[1,3,4], Antti Saraste[1,3,4,8], and Anne Roivainen[1,3,4,5*]

[1]Turku PET Centre, University of Turku, FI-20520 Turku, Finland; [2]Department of Chemistry, University of Turku, FI-20500 Turku, Finland; [3]InFLAMES Research Flagship, University of Turku, FI-20014 Turku, Finland; [4]Turku PET Centre, Turku University Hospital, FI-20520 Turku, Finland; [5]Turku Center for Disease Modeling, University of Turku, FI-20520 Turku, Finland; [6]Accelerator Laboratory, Turku PET Centre, Åbo Akademi University, FI-20520 Turku, Finland; [7]Department of Chemistry, Purdue University, West Lafayette, Indiana, 47907, United States; [8]Heart Centre, Turku University Hospital and University of Turku, FI-20520 Turku, Finland

*Correspondence: Prof. Anne Roivainen, PhD, Turku PET Centre, Kiinamyllynkatu 4-8, FI-20520 Turku, Finland. Tel: +35823132862; Fax: +35822318191; E-mail: anne.roivainen@utu.fi

Pages: 18

Figures: 6

Tables: 6

Table of Contents

Supplementary materials and methods....S-3

Animal model....S-3

Radiosynthesis of [18F]SFB-FOL....S-3

Radiosynthesis of [18F]FOL....S-4

LogD measurements for [18F]SFB-FOL....S-5

Blood analyses....S-5

In vivo PET image analysis....S-6

In vitro experiments....S-6

Supplementary figures....S-7

Figure S1....S-7

Figure S2....S-8

Figure S3....S-9

Figure S4....S-10

Figure S5....S-11

Figure S6....S-12

Table S1....S-13

Table S2....S-14

Table S3....S-15

Table S4....S-16

Table S5....S-17

Table S6....S-18

Supplementary materials and methods

Animal model

Rats under isoflurane anesthesia (4.5% induction, 1.5–2% maintenance) were immunized twice with porcine cardiac myosin (5 mg/mL, M0531; Sigma Aldrich; 0.25 mg dose per rat) in an equal volume of complete Freund's adjuvant: injections were given 1 week apart into the left foot hock. The first immunization was enhanced by intraperitoneal injection of pertussis toxin (250 ng/mL, P2980; Sigma Aldrich; 40 ng of toxin/160 μL per animal). Buprenorphine (0.03 mg/kg subcutaneously) was administered for pain management (twice daily for 2 days after the final immunization).

Radiosynthesis of [^{18}F]SFB-FOL

Using a Sep-Pak Light QMA cartridge (Waters) pre-conditioned with potassium bicarbonate (0.5 M, 2 mL) followed by water (5 mL), [^{18}F]fluoride (3–19 GBq) was eluted into an empty vessel in 2 mL of a Kryptofix/K_2CO_3 solution (diluted in 1 mL of water and 25 mL of acetonitrile). The vessel was sealed, heated to 120ºC, and dried using nitrogen gas. *N*-succinimidyl 4-[^{18}F]fluorobenzoate (SFB) precursor (5 mg) in dry acetonitrile (500 μL) was added to the remaining residue, heated to 100ºC for 15 min, then cooled to 50ºC before adding tetrapropylammonium hydroxide (1 M, 40 μL). To hydrolyze the ^{18}F-intermediate compound, the solution was heated to 120ºC for 6 min in the sealed vessel, dried, and cooled to 50ºC prior to addition of tetramethyl-*O*-(*N*-succinimidyl) uranium tetrafluoroborate (20 mg) in 600 μL of dry acetonitrile. The solution was then heated at 110ºC for 8 min to produce [^{18}F]SFB. Once cooled to 40ºC, the solution was subjected to high-performance liquid chromatography (HPLC) purification on a Jupiter Proteo C18 column (4 µm, 90 Å, 250 × 10 mm, Phenomenex) with UV (λ 254 nm) and radioactivity detection. Gradient elution utilizing 0.1% trifluoroacetic acid (TFA) in water (solvent A) and 0.1% TFA in acetonitrile (solvent B) was performed to separate the product. The elution gradient was set from 27% to 70% B solvent over 14 min, at a flow rate of 4 mL/min. The [^{18}F]SFB product was eluted at 14.5 min and captured in a vessel containing 25 mL of water. This solution was then passed through two tC18

cartridges (Waters) in series, which were pre-equilibrated with ethanol followed by water (5 mL each). The cartridges were then washed with water (5 mL) and [^{18}F]SFB was eluted with acetonitrile (600 μL). To produce [^{18}F]SFB-FOL, a mixture of folate precursor (100 mM, 40 μL) and borate buffer (300 mM, pH 8.6, 460 μL) was added to the [^{18}F]SFB solution, reacted at RT for 10 min, and then neutralized with hydrochloric acid (150 mM, 300 μL). A second HPLC purification was done using a semi-preparative Jupiter Proteo C18 column (4 μm, 90 Å, 250 × 10 mm, Phenomenex) with UV (λ 254 nm) and radioactivity detection, using the same solvents described above. The elution gradient changed from 10% to 30% B over 14 min; [^{18}F]SFB-FOL eluted at approximately 18.5 min. The product was collected in water (25 mL) containing ascorbic acid (2.4 mM), loaded onto a tC18 cartridge (pre-equilibrated as described above), and then eluted with a solution of ethanol (250 μL), water (200 μL), and ascorbic acid (200 mM, 50 μL). The final product was formulated by addition of sterile physiological saline containing ascorbic acid (20 mM, 1.5 mL), with radioactivity concentrations kept below 400 MBq/mL to improve stability and avoid radiolysis. Analytical radio-HPLC using an analytical Jupiter Proteo C18 column (4 μm, 90 Å, 250 × 4.6 mm, Phenomenex) with UV (λ 254 nm) and radioactivity detection confirmed product quality. Using the same solvents mentioned above, a gradient elution was set from 23% to 45% B over 12 min, at a flow rate of 1 mL/min; [^{18}F]SFB-FOL eluted at 7.5 min.

Radiosynthesis of [^{18}F]FOL

[^{18}F]fluoride (3–6 GBq) was eluted from an equilibrated Chromafix PS-HCO_3-45 mg cartridge (Macherey-Nagel) using sterile physiological saline (200–250 μL) and then added to a reaction vessel containing propylene glycol (20 μL), aluminum chloride (2 mM) in sodium acetate buffer (1 M, pH 4.0, 40 μL), acetonitrile (70 μL), and NOTA-folate (200 nmol) and water (50 μL). The mixture was heated at 100°C for 15 min, cooled to 40°C, and then mixed with 800 μL 0.1% TFA. HPLC purification was performed using a semi-preparative Jupiter Proteo C18 column (4 μm, 90 Å, 250 × 10 mm, Phenomenex) with UV (λ 254 nm) and radioactivity detection. The solvents were the same as described above, and the elution gradient was set from 0% B at 1 mL/min for 1 min, increased to 4

mL/min for 4 min, then switched to 30% B solvent over 20 min at a flow rate of 4 mL/min. At 18 min, the product was eluted into water (20 mL), sodium bicarbonate (1 M, 150 µL), and gentisic acid (1 M, 100 µL). It was then loaded onto a tC18 cartridge (Waters, pre-equilibrated with 10 mL ethanol and 10 mL water), washed with water (5 mL), and eluted with 50% ethanol in water (500 µL). The final product was formulated in phosphate-buffered saline (PBS, 2 mL) containing 9% propylene glycol, maintaining the radioactivity concentration below 400 MBq/mL to improve stability and prevent radiolysis. Analytical HPLC was performed (for quality control) using an analytical Jupiter Proteo C18 column (4 µm, 90 Å, 250 × 4.6 mm, Phenomenex) with UV (λ 254 nm) and radioactivity detection, and the solvents mentioned above. The elution gradient ranged from 11% to 25% B over 14 min at a flow rate of 1 mL/min.

Log*D* measurements for [^{18}F]SFB-FOL

The lipophilicity of [^{18}F]SFB-FOL was assessed using 20 kBq of product in PBS (pH 7.4, 600 µL). After adding 1-octanol (600 µL), the mixture was vortexed for 5 min and then centrifuged at 14,100 x *g* for 3 min at RT. Aliquots of the PBS and 1-octanol layers (400 µL each) were analyzed in a γ-counter (1480 Wizard 3"; Perkin Elmer/Wallac), and the 1-octanol-PBS distribution coefficient (Log*D*) was calculated as $\log_{10}$ (counts in 1-octanol/counts in PBS).

Blood analyses

To measure the stability, blood cell binding, and plasma protein binding of both tracers, blood samples (0.2–0.6 mL) were drawn from the tail vein at 10–60 min post-injection. Plasma and blood cells were separated by centrifugation (2,118 × *g* for 5 min at 4°C). An equal volume of acetonitrile was added to the plasma, vortexed, and centrifuged (14,000 × *g* for 2 min at RT) to separate the plasma proteins. The remaining plasma supernatant was filtered through a 0.45 µm Minispike filter (Waters) and adjusted to 1 mL with 0.1% TFA in water for radio-HPLC analysis using a semi-preparative Jupiter Proteo C18 column (4 µm, 90 Å, 250 × 10 nm, Phenomenex) with

UV (λ 254 nm) and radioactivity detection. Using 0.1% TFA in water (solvent A) and 0.1% TFA in acetonitrile (solvent B), the elution gradient for [^{18}F]SFB-FOL was set from 19% to 45% B over 15 min at 5 mL/min. Using the same solvents, the elution gradient for [^{18}F]FOL was set from 8% to 23% B over 14 min, also at 5 mL/min.

In vivo PET image analysis

Carimas 2.10 software (Turku PET Centre, Finland) with the Heart plug-in tool was used to evaluate tracer uptake in the myocardium of rats *in vivo*. Manual orientation of the [^{18}F]FDG images was performed to define the long axis of the myocardium. The myocardial contours were then defined semi-automatically. Co-registration of [^{18}F]FDG images and [^{18}F]SFB-FOL or [^{18}F]FOL images was done by visually aligning the radioactivity of the initial blood pool within the LV cavity with myocardial uptake in the [^{18}F]FDG scans. The myocardial contours from the [^{18}F]FDG images were copied to the co-registered [^{18}F]SFB-FOL or [^{18}F]FOL data. Then, polar maps of [^{18}F]SFB-FOL or [^{18}F]FOL radioactivity in the LV myocardium were generated using matching image orientation and sampling points. Polar map segments corresponding to inflammatory lesions or remote myocardium were confirmed by histology.

In vitro experiments

For the competitive binding studies, slides were pre-incubated in PBS at RT and then incubated in PBS at RT for 4 h with [^{18}F]SFB-FOL alone or in [^{18}F]SFB-FOL with folate glucosamine. After incubation, slides were washed twice with cold PBS, rinsed with cold water, and then dried using a hairdryer before exposure to an imaging plate (BAS-TR2025, Fujifilm) for at least 4 h.

For the cell binding studies, cells were analyzed by flow cytometry using fluorescein isothiocyanate (FITC)-conjugated anti-human FR-β and appropriate isotype control antibodies (mouse IgG-FITC and IgG2a-APC; BioLegend).

Supplementary figures

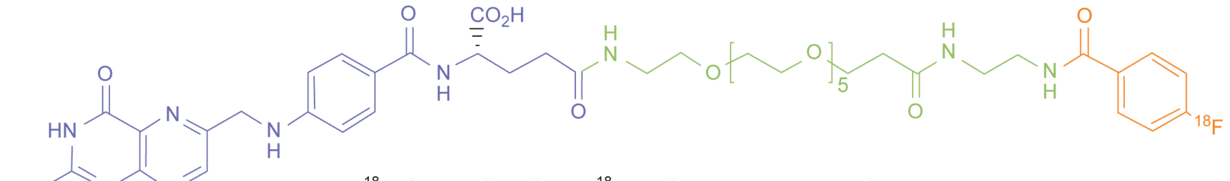

$[^{18}F]$SFB-FOL, $C_{36}H_{54}{}^{18}FN_{10}O_{12}$, MW 818.87 g/mol

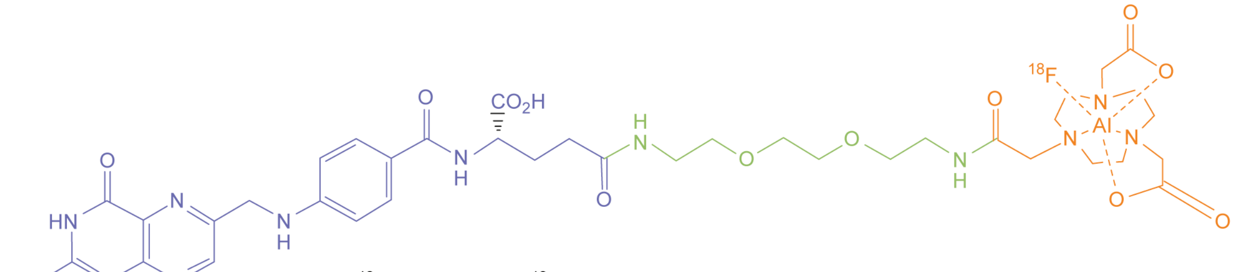

$[^{18}F]$FOL, $C_{37}H_{50}{}^{18}FN_{12}O_{12}$, MW 899.8615 g/mol

Figure S1. Chemical structure of the ^{18}F-labeled folate-based tracers used in the study. Blue: targeting moiety (folate molecule); green: linker; orange: labeling moiety.

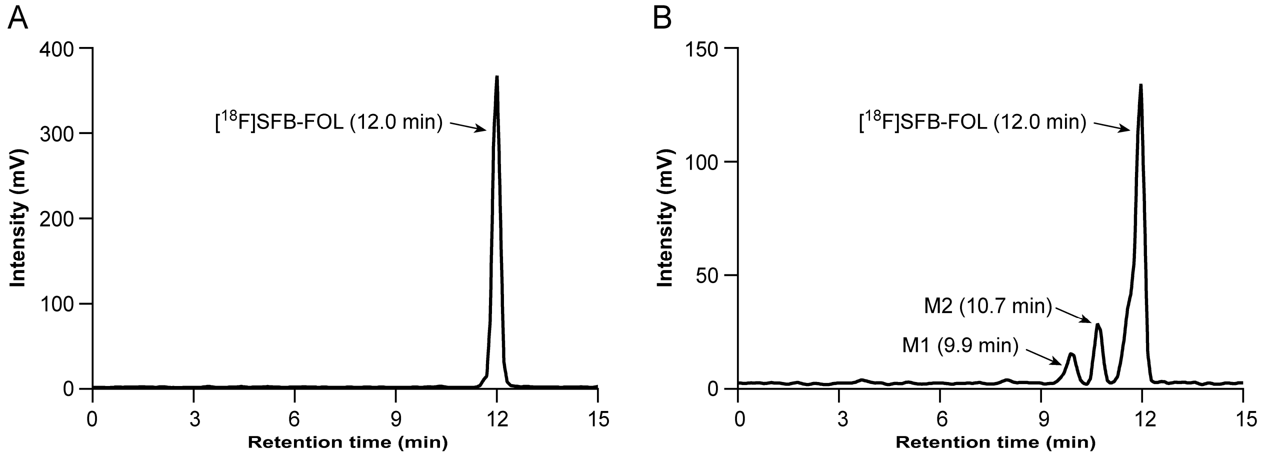

Figgure S2. (A) Representative radiochromatograms of the [^{18}F]SFB-FOL end-product demonstrating 100% [^{18}F]SFB-FOL purity. (B) Representative radiochromatograms of myocarditis rat plasma supernatant, taken 70 min after injection of [^{18}F]SFB-FOL; [^{18}F]SFB-FOL (72.3% pure) and two radioactive metabolites (M1 and M2) can be seen.

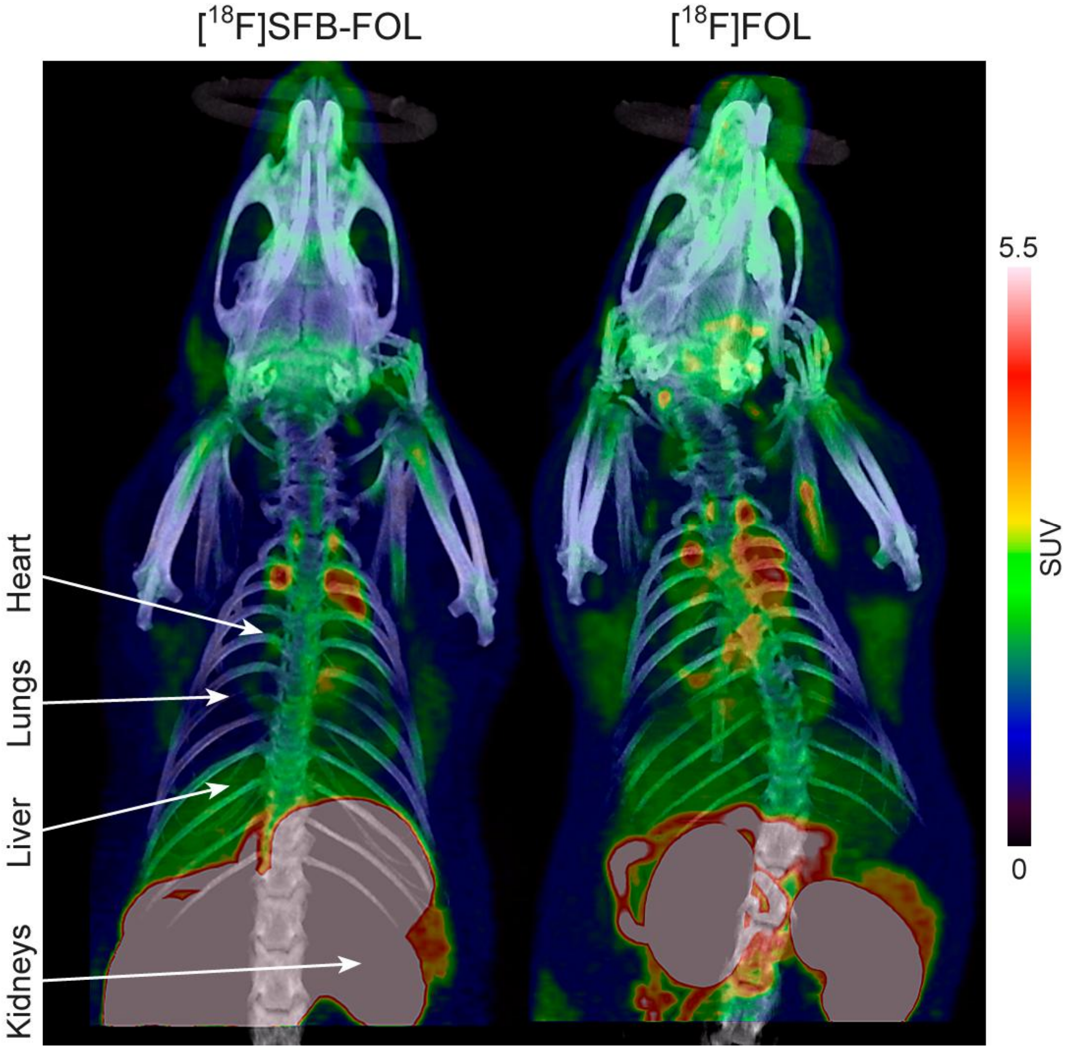

Figure S3. Representative coronal *in vivo* PET images of both tracers in the same animal.

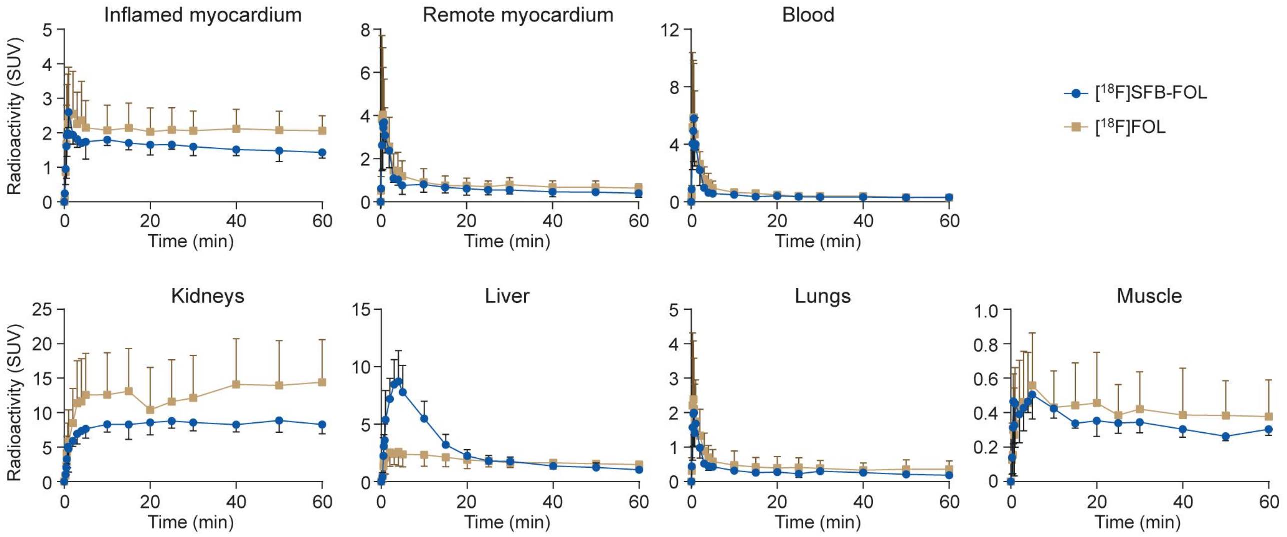

Figure S4. Time-activity curves for regions of interest in immunized rats after injection of folate tracers.

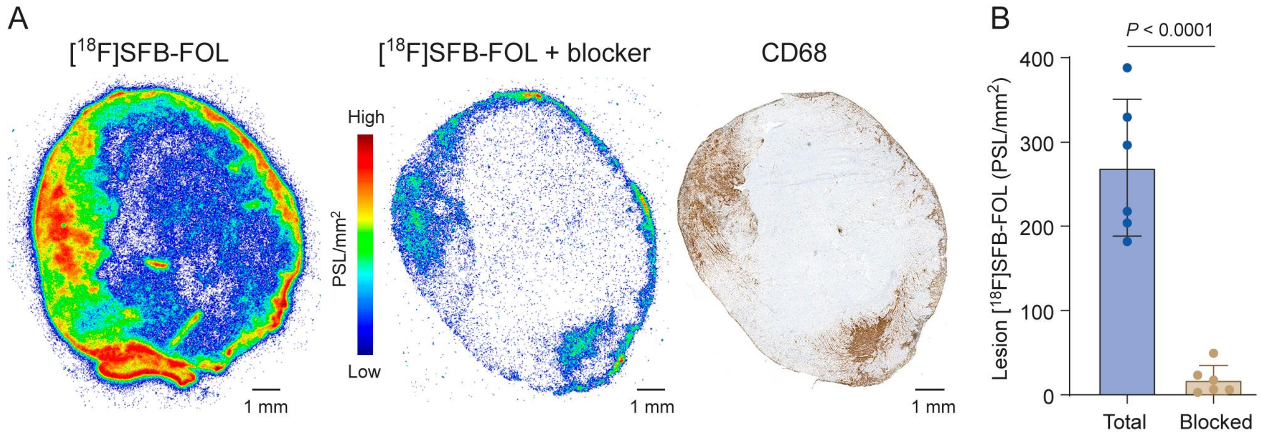

Figure S5. *In vitro* competitive binding assay for [^{18}F]SFB-FOL with a folate glucosamine blocker in sections of myocardium obtained from rats at 21 days post-immunization. (A) Representative autoradiographs and anti-CD68 immunohistochemical staining of [^{18}F]SFB-FOL. (B) Quantification of [^{18}F]SFB-FOL binding *in vitro*. PSL/mm^2 = photostimulated luminescence per mm^2.

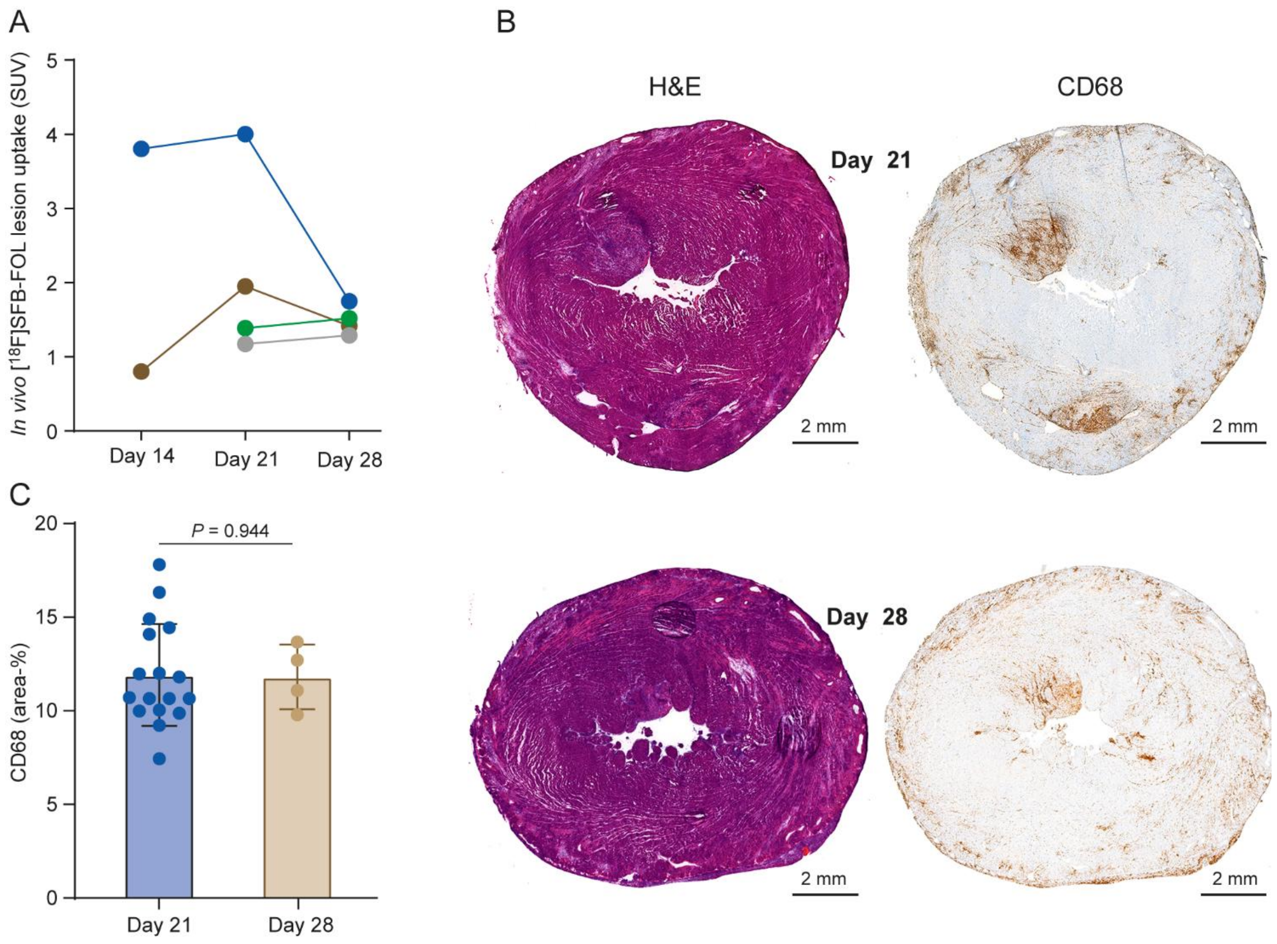

Figure S6. (A) Standardized uptake values (SUV) for [^{18}F]SFB-FOL in rat myocarditis lesions over time. (B) Representative histology images (H&E and immunohistochemical staining with an anti-CD68 antibody) obtained at Day 21 and Day 28 post-immunization. (C) Quantification of CD68-positivity (area-%) on Day 21 *vs.* Day 28 post-immunization.

Table S1. *Ex vivo* biodistribution of tracers in healthy Lewis rats at 70 min post-injection

Tissue	[18F]SFB-FOL	[18F]FOL	*P* value
Adipose tissue, brown	0.12 ± 0.03	0.21 ± 0.02	0.0003
Adipose tissue, white	0.04 ± 0.01	0.08 ± 0.01	0.070
Adrenal glands	0.11 ± 0.03	0.22 ± 0.02	0.009
Blood	0.01 ± 0.01	0.05 ± 0.02	0.602
Bone marrow	0.57 ± 0.10	0.72 ± 0.10	0.002
Bone, femur	0.15 ± 0.03	0.22 ± 0.02	0.0004
Bone, skull	0.05 ± 0.01	0.08 ± 0.01	0.023
Brain	0.01 ± 0.003	0.01 ± 0.00x	0.028
Heart	0.06 ± 0.01	0.15 ± 0.02	0.001
Intestine, large	0.17 ± 0.03	0.26 ± 0.02	0.030
Intestine, small	0.82 ± 0.26	0.38 ± 0.13	0.004
Kidneys	2.71 ± 1.30	8.00 ± 3.48	0.008
Liver	0.10 ± 0.02	0.17 ± 0.02	0.093
Lungs	0.23 ± 0.32	0.14 ± 0.01	0.002
Lymph node	0.62 ± 0.11	1.13 ± 0.14	0.016
Muscle	0.06 ± 0.01	0.12 ± 0.02	0.00002
Pancreas	0.09 ± 0.02	0.16 ± 0.01	0.683
Salivary glands	0.29 ± 0.06	0.50 ± 0.05	0.001
Spleen	0.30 ± 0.06	0.42 ± 0.05	0.0003
Testes	0.05 ± 0.01	0.10 ± 0.01	0.002
Thymus	0.11 ± 0.02	0.19 ± 0.04	0.003
Urine	1.09 ± 0.73	1.38 ± 0.91	0.006

Results are expressed as the percentage of injected radioactivity dose per gram of tissue (mean ± SD, *n* = 5 [18F]SFB-FOL, *n* = 4 [18F]FOL). *P* values were calculated using Student's *t* test.

Table S2. *Ex vivo* biodistribution of [^{18}F]SFB-FOL in control and immunized rats at 70 min post-injection

Tissue	Controls	Immunized	*P* value
Adipose tissue, brown	0.12 ± 0.03	0.25 ± 0.24	0.279
Adipose tissue, white	0.04 ± 0.01	0.13 ± 0.05	0.005
Adrenal glands	0.11 ± 0.03	0.17 ± 0.07	0.136
Blood	0.01 ± 0.01	0.01 ± 0.01	0.190
Bone marrow	0.57 ± 0.10	0.79 ± 0.43	0.337
Bone, femur	0.15 ± 0.03	0.26 ± 0.10	0.047
Bone, skull	0.05 ± 0.01	0.09 ± 0.05	0.150
Brain	0.01 ± 0.003	0.01 ± 0.003	0.288
Heart	0.06 ± 0.01	0.50 ± 0.26	0.006
Intestine, large	0.17 ± 0.03	0.28 ± 0.10	0.035
Intestine, small	0.82 ± 0.26	0.77 ± 0.24	0.755
Kidney	2.70 ± 1.30	4.16 ± 1.20	0.085
Liver	0.10 ± 0.02	0.37 ± 0.16	0.006
Lung	0.23 ± 0.32	0.20 ± 0.07	0.785
Lymph node	0.62 ± 0.11	0.74 ± 0.41	0.569
Muscle	0.06 ± 0.01	0.07 ± 0.02	0.082
Pancreas	0.09 ± 0.02	0.19 ± 0.09	0.050
Salivary glands	0.29 ± 0.06	0.69 ± 0.39	0.056
Spleen	0.30 ± 0.06	1.07 ± 0.41	0.003
Testes	0.04 ± 0.01	0.04 ± 0.01	0.254
Thymus	0.11 ± 0.02	0.58 ± 0.24	0.002
Urine	1.09 ± 0.73	3.41 ± 2.73	0.158

Immunized rats were sacrificed 21 days after initial immunization. Results are expressed as the percentage of injected radioactivity dose per gram of tissue (mean ± SD, n = 5 healthy controls, n = 7 immunized rats). P values were calculated using Student's t test.

Table S3. *Ex vivo* biodistribution of tracers in immunized rats at 70 min post-injection

Tissue	[18F]SFB-FOL	[18F]FOL	*P* value
Adipose tissue, brown	0.25 ± 0.24	0.36 ± 0.23	0.571
Adipose tissue, white	0.13 ± 0.05	0.16 ± 0.12	0.595
Adrenal glands	0.17 ± 0.07	0.36 ± 0.08	0.005
Blood	0.01 ± 0.00x	0.04 ± 0.05	0.150
Bone marrow	0.79 ± 0.43	1.20 ± 0.53	0.232
Bone, femur	0.26 ± 0.10	0.44 ± 0.04	0.008
Bone, skull	0.09 ± 0.05	0.16 ± 0.04	0.049
Brain	0.01 ± 0.00x	0.01 ± 0.00x	0.082
Heart	0.50 ± 0.26	0.84 ± 0.16	0.089
Intestine, large	0.28 ± 0.10	0.46 ± 0.11	0.031
Intestine, small	0.77 ± 0.24	0.73 ± 0.07	0.791
Kidneys	4.16 ± 1.20	9.04 ± 0.56	0.00004
Liver	0.37 ± 0.16	0.97 ± 0.24	0.0009
Lungs	0.20 ± 0.07	0.32 ± 0.04	0.013
Lymph node	0.74 ± 0.41	1.10 ± 0.11	0.141
Muscle	0.07 ± 0.02	0.13 ± 0.02	0.001
Pancreas	0.19 ± 0.09	0.49 ± 0.15	0.003
Salivary glands	0.69 ± 0.39	1.12 ± 0.27	0.101
Spleen	1.07 ± 0.41	2.05 ± 0.49	0.008
Testes	0.04 ± 0.01	0.07 ± 0.01	0.002
Thymus	0.58 ± 0.24	0.89 ± 0.19	0.061
Urine	3.41 ± 2.73	2.16 ± 0.94	0.435

Rats were sacrificed 21 days after initial immunization. Results are expressed as the percentage of injected radioactivity dose per gram of tissue (mean ± SD, *n* = 7 [18F]SFB-FOL, *n* = 4 [18F]FOL). *P* values were calculated using Student's *t* test.

Table S4. Effect of blocking on *ex vivo* biodistribution of [^{18}F]SFB-FOL in immunized rats at 70 min post-injection

Tissue	Total	Blocked	*P* value	Effect	Change (%)
Adipose tissue, brown	0.25 ± 0.24	0.06 ± 0.03	0.084	↓	78.18
Adipose tissue, white	0.13 ± 0.05	0.09 ± 0.17	0.527	↓	33.41
Adrenal glands	0.17 ± 0.07	0.06 ± 0.07	0.023	↓	63.32
Blood	0.01 ± 0.00	0.01 ± 0.01	0.771	↑	11.95
Bone marrow	0.79 ± 0.43	0.10 ± 0.09	0.002	↓	86.87
Bone, femur	0.26 ± 0.10	0.04 ± 0.03	0.0001	↓	86.10
Bone, skull	0.09 ± 0.05	0.01 ± 0.01	0.005	↓	84.47
Brain	0.01 ± 0.00	0.00 ± 0.00	0.088	↓	43.89
Heart	0.50 ± 0.26	0.08 ± 0.06	0.002	↓	84.10
Intestine, large	0.28 ± 0.10	0.20 ± 0.37	0.570	↓	30.28
Intestine, small	0.77 ± 0.24	0.36 ± 0.24	0.012	↓	53.35
Kidneys	4.16 ± 1.20	2.93 ± 0.82	0.053	↓	29.58
Liver	0.37 ± 0.16	0.19 ± 0.09	0.040	↓	46.86
Lungs	0.20 ± 0.07	0.03 ± 0.02	< 0.0001	↓	84.14
Lymph node	0.74 ± 0.41	0.27 ± 0.17	0.021	↓	63.45
Muscle	0.07 ± 0.02	0.03 ± 0.02	0.002	↓	63.57
Pancreas	0.19 ± 0.09	0.04 ± 0.04	0.003	↓	76.61
Salivary glands	0.69 ± 0.39	0.15 ± 0.12	0.006	↓	77.62
Spleen	1.07 ± 0.41	0.12 ± 0.10	0.0001	↓	88.91
Testes	0.04 ± 0.01	0.03 ± 0.02	0.630	↓	12.76
Thymus	0.58 ± 0.24	0.19 ± 0.20	0.007	↓	67.47
Urine	3.41 ± 2.73	6.98 ± 3.40	0.056	↑	104.45

Results are expressed as the percentage of injected radioactivity dose per gram of tissue (mean ± SD, n = 7 Total, n = 7 Blocked). *P* values were calculated using Student's *t* test. ↓ = decrease, ↑ = increase.

Table S5. *In vivo* uptake of [^{18}F]SFB-FOL in the myocardium and adjacent tissues in the same rats at 20–40 min post-injection

Region of interest	[^{18}F]SFB-FOL Day 14*	[^{18}F]SFB-FOL Day 21	[^{18}F]SFB-FOL Day 28
Lesion	1.8 ± 1.1	1.8 ± 0.8	1.4 ± 0.4
Remote	1.3 ± 0.6	1.5 ± 1.0	0.9 ± 0.3
Blood†	0.3 ± 0.1	0.4 ± 0.0	0.3 ± 0.1
Liver	1.7 ± 0.9	2.0 ± 0.9	1.7 ± 0.8
Lung	0.3 ± 0.1	0.3 ± 0.1	0.3 ± 0.1

Results are expressed as standardized uptake values (mean ± SD, $n = 4$). $P > 0.05$ (for all comparisons), calculated using a mixed-effects model for repeated measures. *$n = 2$, †from the left ventricle.

Table S6. *Ex vivo* biodistribution of [^{18}F]SFB-FOL in immunized rats at 70 min post-injection

Tissue	Day 21	Day 28	*P* value
Adipose tissue, brown	0.25 ± 0.24	0.14 ± 0.07	0.424
Adipose tissue, white	0.13 ± 0.05	0.07 ± 0.03	0.078
Adrenal glands	0.17 ± 0.07	0.13 ± 0.07	0.420
Blood	0.01 ± 0.00	0.01 ± 0.01	0.662
Bone marrow	0.79 ± 0.43	0.63 ± 0.19	0.532
Bone, femur	0.26 ± 0.10	0.21 ± 0.06	0.447
Bone, skull	0.09 ± 0.05	0.04 ± 0.01	0.151
Brain	0.01 ± 0.00	0.01 ± 0.00	0.420
Heart	0.50 ± 0.26	0.37 ± 0.17	0.430
Intestine, large	0.28 ± 0.10	0.19 ± 0.08	0.172
Intestine, small	0.77 ± 0.24	0.52 ± 0.42	0.286
Kidneys	4.16 ± 1.20	4.95 ± 0.80	0.301
Liver	0.37 ± 0.16	0.27 ± 0.17	0.411
Lungs	0.20 ± 0.07	0.14 ± 0.07	0.229
Lymph node	0.74 ± 0.41	1.04 ± 0.35	0.280
Muscle	0.07 ± 0.02	0.06 ± 0.01	0.154
Pancreas	0.19 ± 0.09	0.12 ± 0.07	0.283
Salivary glands	0.69 ± 0.39	0.39 ± 0.16	0.196
Spleen	1.07 ± 0.41	0.85 ± 0.59	0.507
Testes	0.04 ± 0.01	0.03 ± 0.00	0.393
Thymus	0.58 ± 0.24	0.31 ± 0.12	0.082
Urine	3.41 ± 2.73	2.03 ± 1.51	0.409

Results are expressed as the percentage of injected radioactivity dose per gram of tissue (mean ± SD). *P* values were calculated using Student's t test. Day 21, n = 7; Day 28, n = 4.
